# New York Exhibition of the Industry of All Nations

**Published:** 1854-01

**Authors:** 


### Selected Articles.

### *New York Exhibition of the Industry of all Nations.*

We have taken some pains to examine the specimens of Dental Operations and Dental Mechanics, now on exhibition at the "World's Fair" in New York. We find in the catalogue, about twenty-two cases (including dental instruments), in the United States department, and one from England; but after making diligent search for them, during several visits, there are still some which we bave not been able to find. Most of those that we have seen, were made for show and not for service in the mouth, and, of course, are not fair specimens of the kind of workmanship executed by the exhitors, for their patients and customers. Very little good can result from exhibitions of this kind, unless to the pockets of the exhibitors, and we should think, that this method of advertising had been practised in the fairs of the American Institute, and other similar exhibitions too often to pay. If the expert and skillful members of our profession, would unite their efforts, and make an exhibition of dental mechanics and operations, which had been constructed for service in the mouths of their patrons and patients; such, for instance, as gold plates and palates which had been worn in the mouth, gold fillings which had seen some years service before the teeth were extracted, apparatus for regulating teeth, with models taken before and after its application, instruments of practical utility, and such other workmanship as is practical and useful; we can understand how such an exhibition would raise the standard of dental mechanics, and redound greatly to the advantage of the public and the profession.

But we have seen enough of *show pieces*, galvanised silver or copper plates, fitted to carved models, gold fillings put in while the teeth were securely screwed into a bench-vice, and finished with a buff or brush wheel, with rouge and buckskin, may be pleasing to the eye of the uninitiated, and serve to draw some cheap customer to the office of the dentist, after the exhibition has closed, but they can never raise the standard of genuine dental operations, nor elevate the calling of the Dental Surgeon in the estimation of sensible and discerning men. We regret to say, that there are too many cases of this kind on *show* at the great exhibition of the industry of all nations; but we will proceed to notice such as we have seen, and leave those of our readers who have not had an opportunity to witness the specimens themselves, to judge of the correctness of our views.

Robert A. Porter, of Philadelphia, exhibits a number of tolerably good blocks. The principal feature in them is the imitation of carious spots in the teeth. In some of them there are poor gold fillings ; but it is difficult to put good ones into smooth glazed cavities in mineral teeth. This case also contains two silver plates with teeth mounted on them ; one is an ordinary clasped piece, and the other is a cavity plate for a partial set. There is nothing remarkably good in the case, though the whole is fair.

S. Brown, New York, has a small case, exhibiting Stockton's teeth, and "Reynolds Dental Detergen." The assortment of teeth in this case is small, and it illustrates the saying that, "When a man's name is up he may lay in bed 'till noon."

Jones, White & McCurdy, of Philadelphia and New York, have a large assortment of their teeth there, embracing gum, pivot and plain plate teeth; they are neatly arranged, and so as to show the peculiar qualities of their teeth to advantage. There are also in this case specimens of corrundum wheels, slabs and files, Morson's beautiful forceps, and Dr. L. S. Parmly's apparatus for cleaning the teeth, and various dental articles from their furnishing stores.

Warren Rowell exhibits a duplicate specimen of palate plate, containing artificial teeth, which was "invented and constructed by himself," and has been worn since 1841. A description of this ingenious piece of mechanism, can be found in Harris' Dental Surgery.

Charles F. Mermier, exhibits a small case, intended to show the various steps in the construction of a set of artificial teeth, from the taking of the impression to the finishing. The wax impression is shown, the metallic casting, the plate, the blocks, in biscuit and after burning; but the whole are mixed up together, and without any description, so that a novice can hardly understand what the exhibitor intends to show. The blocks are tolerably good.

Doctors Chapman, of New York. These gentlemen exhibit principally block work, neatly and ingeniously arranged in a beautiful case. The principal feature in it consists of a double set having a movable lower casting, to show the antagonising of the teeth. By a simple contrivance, the teeth are made to open and shut. The direction on the case being to "turn the silver knob, and notice the closing of the teeth, and then raise the wooden one and *take our card.*" This set is very well made, but the blocks would be improved by giving more transluscency to the teeth. The gum is also, either too deeply colored or too bricky, and they have not been quite careful enough in joining their blocks.

Ambler & Avery, of New York. Most of the specimens of this case, have been exhibited at the fair of the American Institute, where the exhibitors obtained a gold medal; some of them were also in the Crystal Palace at London. There is some excellent workmanship in this case; among the pieces, we recognised one, made by Avery, consisting of two lateral incisors, on a cavity plate which we saw worn in the mouth, and then pronounced a beautiful piece of workmanship. In this case, there are also several fancy pieces.

James Fowler, of New York. In this case there are five full upper sets, most, if not all, made for exhibition, and all showing excellent workmanship.

John Allen, of Cincinnati. Dr. Allen exhibits specimens of his new style of continuous gums fused upon platina plates, together with some of the old style of plain single teeth upon gold plates, for the purpose of contrasting the two methods. Instead of an ordinary, old fashioned set of teeth, if Dr. Allen had placed by the side of his new style of work, a handsome piece of Crofoot's or Porter's blocks, mounted on gold plates by either of these gentlemen, the contrast would have been much less favorable to the new style, and the exhibition would have been much fairer, as it would have shown the improvements which other dentists have made, as well as those of the exhibitor.

The specimens of "continuous gums" are well executed and show Dr. Allen's improvement to great advantage.

H. B. Hale of Boston. This case contains an upper set of natural teeth united by a wax base, most of the teeth are filled with gold. As far as we could judge from appearance, these teeth were filled after they were extracted, for exhibition. The fillings appeared to be well put in, and were hansomely finished. Such work, if done in the mouth, we should pronounce good.

R. T. Reynolds, of Philadelphia. This is a case of excellent workmanship, containing one full upper set of blocks, one double set of single gum teeth remarkably well ground together, one upper set of common plate teeth well fitted to the plate, and one clasp case, the clasps embracing the second molars. The last was too heavy for clasps, and should have been made, we think, to be worn by atmospheric pressure; but the workmanship of the whole is admirable.

T. Palmer, of Fitchburgh, Mass. Whether this case is exhibited for the purpose of exciting patriotic feeling in the American people, or advancing the science of humbug, we will not decide. It is one of the largest in the exhibition, and has, perhaps, as little in it to attract the attention of dentists as the smallest case there. It is surmounted by the American eagle, bearing in his beak an upper set of teeth, and flanked by the stars and stripes. There are a number o blocks containing each an entire set, burned in one piece, (o course for show) some of them mounted. Two or three cases of block work are mounted on galvanized plates, and may be duplicates of cases made for the mouth. There is also quantity of mouth wash and tooth paste in the case.

Ballard & Kingsley of New York. This is the only case of real practical work which we saw in the exhibition; most of the cases having been worn for months, and loaned by the owners for exhibition. There is one piece, which we saw worn in the mouth, in which the teeth are very well colored to represent the dark stains so frequently seen across the upper part of front teeth. Two full upper sets, chamber plates, very well done. One chamber plate, with only the lateral incisors. Another chamber plate contains a block on one side extending from the lateral incisor to the first molar, and made to supply the loss of a large portion of the jaw bone. Several specimens of blocks, not mounted, but duplicates of those now worn in the mouth. Among the fancy pieces in this case, is one showing denuded fangs; very natural. A block representing a crowded denture, is a fine specimen of carving. The dental mechanism in this case, was all constructed by Mr. Kingsley. There are also, several specimens of gold fillings in natural teeth, by Dr. Ballard, which are very beautiful; but done out of the mouth.

The above embraces all the work in dental mechanics, which we were able to find, though several more are put down in the catalogue. In the French department, we noticed several cases of surgical instruments of rare beauty; one in particular, by Luer, surpasses anything of the kind which we ever saw.

